# The ABC of academic procrastination: Functional analysis of a detrimental habit

**DOI:** 10.3389/fpsyg.2022.1019261

**Published:** 2022-11-03

**Authors:** Frode Svartdal, Jon Arne Løkke

**Affiliations:** ^1^Department of Psychology UiT The Arctic University of Norway, Tromsø, Norway; ^2^Department of Welfare, Management and Organisation, Østfold University College, Halden, Østfold, Norway

**Keywords:** procrastination, functional analysis, intervention, prevention, self-control

## Abstract

Academic procrastination – habitually delaying work with academic tasks to the extent that the delays become detrimental to performance, wellbeing, and health – represents a substantial personal, systemic, and societal problem. Still, efforts to prevent and reduce it are surprisingly scarce and often offered as treatment regimens rather than preventive efforts. Based on the principles of functional analysis and a broad examination of factors that are important for academic procrastinatory behaviors, this paper aims to describe a strategy for analyzing individual controlling conditions for procrastination and give parallel advice on how to change those controlling conditions. Both are ideographic, allowing for individual and dynamic analyses of factors responsible for instigating and maintaining procrastination, as well as tailor-made remedies that address controlling conditions in preventive and curative efforts to reduce procrastination. Although functional analysis integrates well with important research findings in the procrastination field, this approach suggests new criteria for identifying procrastinatory behaviors and an alternative model for analyzing their control conditions. We conclude that a functional approach may supplement procrastination research and efforts to prevent and alleviate this detrimental habit.

## Introduction

Procrastination – delaying tasks despite expecting to be worse off for the delay (Steel, 2007) – is a common problem among students. Conservative estimates indicate a prevalence of at least 50%, suggesting that half or more of all students habitually procrastinate tasks such as reading before tests and exams and preparing assignments (e.g., Pychyl et al., 2000; Schouwenburg et al., 2004). Academic performance, health, and wellbeing are negatively affected (e.g., Tice and Baumeister, 1997; Steel, 2007; Kim and Seo, 2015), making academic procrastination a pervasive problem that puts many students in a disadvantageous position and represents considerable costs at the individual, institutional, and societal levels (e.g., Steel et al., 2018). Effective preventive and curative measures are imperative.

A proper understanding of procrastination, in terms of definitional criteria and underlying mechanisms, is necessary to grasp the complexity of the procrastination phenomenon when developing preventive and curative measures. Researchers agree that behavioral delay is a fundamental criterion for procrastination. Notably, modern definitions emphasize that such delays must be detrimental for the person to be regarded as procrastination (e.g., Steel, 2007; Ferrari, 2010; Klingsieck, 2013). Thus, procrastination occurs when planned work is delayed, with negative consequences as a foreseeable result (Steel, 2007). Examining the nomological network of procrastination, van Eerde (2003a) demonstrated that procrastination correlates positively with other negative states and negatively with positive states. Hence, when measuring procrastination, it is necessary to separate procrastination from strategic delay (e.g., Klingsieck, 2013) and other forms of reasonable delay (e.g., delay submitting your thesis because your advisor advised you to rewrite the discussion part). Unfortunately, separating procrastinatory behavioral delays from other forms of behavioral delays has proven complicated. In the absence of suitable criteria, researchers have often resorted to behavioral delay in general as a proxy for behavioral procrastination (e.g., Miyake and Kane, 2021; Vangsness et al., 2022). Even self-report scales measuring procrastination struggle to separate rational delays from suboptimal or irrational delays (e.g., Svartdal and Steel, 2017; Svartdal and Nemtcan, 2022), resulting in scale scores that include non-procrastinatory delays and thus compromise their validity.

As for mechanisms, procrastination is often understood as a breakdown in self-regulation (e.g., Steel, 2007). Self-regulation is itself a complex construct (e.g., Inzlicht et al., 2020). In the case of procrastination, four general self-regulatory models are particularly relevant. First, in a dual-process model of self-control, procrastination results from an automatic, non-reflective impulse system “winning” over a more rational and effortful reflective system (e.g., Hofmann et al., 2009; Heatherton and Wagner, 2011). Impulsiveness is closely related to breakdown in goal-management and procrastination (e.g., Gustavson et al., 2014). Repeated wins of the impulse system seem to be a characteristic of procrastination, as goal-directed behaviors are replaced by non-functional behaviors, resulting in delayed planned work. Second, a model understanding procrastination resulting from dysfunctional emotional regulation has gained considerable momentum (e.g., Sirois and Pychyl, 2013; Sirois and Kitner, 2015). Negative emotion, for example, perceived task aversiveness, is a strong predictor of procrastination (e.g., Steel, 2007). Emotion regulation takes precedence over self-regulation towards long-term goals when tasks associated with negative emotions (e.g., frustration, boredom, negative affect) are delayed or avoided. Procrastinatory behaviors under such conditions produce immediate and short-term mood repair, making such behaviors “adaptive” in the short-term perspective. However, since they disrupt planned work, these behaviors are “irrational” and maladaptive, given a long-term goal. Third, the limited resource model of self-regulation (e.g., Baumeister and Heatherton, 1996) may also be relevant for understanding the breakdowns in self-regulation seen in procrastination. Notably, given the strong correlation between lack of energy and procrastination, *r* = 0.60 (Steel et al., 2018), it is likely that low energy is associated with delayed behavioral onset as well as with less efficient goal striving. A fourth model may supplement the models discussed and maybe change focus. Growing evidence indicates that successful self-regulation towards a goal is not necessarily related to effortful inhibition of the impulse system, as research demonstrates that individuals high in trait self-control and conscientiousness engage in *less* self-control in situations with temptations and other situational challenges (Hofmann and Kotabe, 2012; Milyavskaya and Inzlicht, 2017; Grund and Carstens, 2019; Hennecke and Bürgler, 2020; Inzlicht et al., 2020). These individuals seem to use preventive self-control strategies that help them avoid dealing with temptations and other situational challenges – they do not encounter them. For example, Duckworth et al. (2016) demonstrated how the individual applies strategies proactively by choosing or changing situations to weaken undesirable impulses and potentiate desirable ones. As procrastinators are present-oriented and less apt to simulate future situations concretely in future episodic thinking (e.g., Sirois and Pychyl, 2013; Rebetez et al., 2016), they may be handicapped in such proactive self-regulative efforts, making this model potentially relevant for procrastination.

Little is known about the relative importance of these (or other) forms of self-regulation in procrastination. In addition, as self-regulation focuses on strategies initiated by the individual, other factors that challenge goal-directed behavior should be examined. In the academic context, “procrastination-friendly” situational and organizational factors are of particular interest (e.g., Nordby et al., 2017; Svartdal et al., 2020; Baars et al., 2021). Clearly, such factors (e.g., long deadlines, a large degree of freedom, and lack of structure in the study situation) are invitations to delay unnecessarily and may counteract self-regulatory efforts.

Attempts to prevent and reduce procrastination include advice and web-based information, as well as treatment efforts to help students overcome more serious procrastination problems. A meta-analysis of 24 intervention studies (van Eerde and Klingsieck, 2018) found Cognitive Behavior Therapy (CBT) to demonstrate the most promising effects. Possible moderator variables, such as the duration of the intervention, had no significant effects. Similar support for CBT was found in another meta-analysis by Rozental et al. (2018). CBT can be seen as an approach that addresses the reflective system in self-regulation. Thus, in procrastination interventions, the focus is on correcting dysfunctional and irrational thoughts, improving prioritization and goal-setting skills, and training students in self-monitoring and stimulus control techniques (see van Eerde and Klingsieck, 2018). Unfortunately, CBT interventions are relatively costly and require a high degree of expertise to be implemented. Furthermore, such interventions are primarily aimed at individuals already suffering from chronic procrastination (e.g., Rozental et al., 2015). Hence, intervention efforts with lower expertise and cost thresholds, as well as the possibility to work in a preventive manner, are preferable. Interventions, including CBT, are also generic in nature, as they focus on sets of skills assumed to be important to prevent or combat procrastination. In an important paper, Steel and Klingsieck (2016) identified a common factor (i.e., conscientiousness and its facets, such as self-discipline and impulsiveness) as important for all procrastinators. However, after controlling for conscientiousness, these authors found students to procrastinate for different reasons. For example, some may procrastinate for social reasons (those high in extraversion), whereas others put off because of anxiety (those high in neuroticism). These results indicate common characteristics of all procrastinators as well as individual differences, depending on personality characteristics or personal history. An important implication of these findings is that interventions should be customized to fit individual profiles – “one size does not fit all” (Steel et al., 2021, p. 16). Steel and Klingsieck (2016) also argued that common explanations of procrastination in terms of neuroticism apply only to a small part of all procrastinators and that factors related to willpower (e.g., impulsiveness and self-discipline) should “be recommended as being the backbone of all interventions with all forms or typologies of procrastination” (p. 43).

In summary, these considerations indicate that multiple challenges face the procrastination research field. In terms of definition, understanding of mechanisms, and prevention/treatment efforts, a need for a fresh look is indicated.^1^ Hence, we discuss an approach that has proven helpful in understanding, alleviating, and preventing problematic behavior and that also has been demonstrated to have comparable outcome results to CBT. Specifically, we explore the utility of functional analysis (FA) in assessing and changing procrastinatory behaviors. In FA (e.g., Hanley et al., 2003; Magidson et al., 2014; Fisher et al., 2021), the target behavior (in this case, procrastinatory behavior) is assessed at the individual level in terms of a three-term contingency model (ABC): *Antecedent stimuli* (A) or under which conditions or antecedents do procrastinatory behaviors occur? *Behavior* (B) or what are the behaviors involved in procrastination? *Consequences* (C) or what are the typical immediate consequences of such procrastinatory behaviors? The purpose of this analysis is to determine controlling conditions: Why do procrastinatory behaviors occur, and how may controlling conditions be changed?

The ABC approach encompasses a broad set of principles derived from the psychology of learning, including stimulus control, reinforcement, extinction, and many others (e.g., Bellack et al., 2012; Mazur, 2017; Rachlin et al., 2018). Based on these principles, the ABC model has proven to shed light on how problematic behaviors develop and are maintained and how problems may be alleviated by systematically changing the controlling factors. This approach has been widely used in behavior modification (e.g., Magidson et al., 2014) as well as in settings relevant to procrastination, for example, in organizations (e.g., Wilder et al., 2009) and schools (e.g., Vargas, 2013). Notably, in the analysis and treatment of depression, a method based on functional analysis, the behavioral activation for depression model (e.g., Veale, 2008), has been found to be a viable approach, and randomized clinical trials have documented this approach to be comparable to CBT (Cuijpers et al., 2007; Dimidjian et al., 2014; Simmonds-Buckley et al., 2019; Stein et al., 2020). Martell et al. (2022) provide an in-depth clinical overview of this approach. Unfortunately, except for a few published studies that at least in part rely on this method (see Gresham et al., 1999; Tuckman and Schouwenburg, 2004), functional analysis is surprisingly little used in procrastination research and interventions. One recent exception is a study focusing on bedtime procrastination (Suh et al., 2021). Bedtime procrastination is “failing to go to bed at the intended time, while no external circumstances prevent a person from doing so” (Kroese et al., 2014). Suh et al. developed a four-session structured intervention that applied some of the procedures suggested in the present paper. For example, they assessed the main function of bedtime procrastination with an emphasis on the emotional and behavioral functions this behavior serves for the individual, and participants were helped to find alternative behaviors to bedtime procrastination that served the same function. Results were positive, with a significant reduction in bedtime procrastination both at post-test and follow-up.

## Plan for this paper

In the sections to follow, we first present a brief overview of the basics of functional analysis as applied to procrastination, followed by a discussion of how this approach differs from established approaches. Then we examine, in some detail, how the rich field of procrastination will fit in an analysis in terms of A (Antecedent conditions), B (Behavior), and C (Consequences). As will be shown, the fit is surprisingly good, indicating that FA indeed presents itself as a promising approach to supplement traditional approaches. However, differences in operationaliations of procrastinatory behaviors as well in the way such behaviors are analyzed indicate important contributions of FA.

### Functional analysis of procrastination

FA assumes that behavior – procrastinatory behavior included – is adaptive and, to a large degree, learned and hence modifiable. However, sometimes adaptation to a given environment goes astray. For example, fear of wasps may be functional, but in phobic reactions to wasps, the behavior is exaggerated and often maladaptive. In the case of (academic) procrastination, the main problem is that some goal-relevant behavior (e.g., academic work) is replaced by other activities too often (e.g., watching TV), resulting in a delay in goal-relevant behaviors. Whereas procrastination research has focused primarily on delayed goal-relevant activity, FA also examines the “irrational” procrastinatory behaviors and asks why these are preferred.

As noted, FA assesses procrastinatory behaviors at the individual level in terms of a three-term contingency analysis. The dynamic interplays between the ABCs represent the very heart of functional analysis, as the Cs following Bs are assumed to work as a causal factor. Hence, if you choose to respond to an aversive task (A) with an avoidance behavior (B), and that behavior then is typically followed by stress reduction and improved mood (C), those consequences work to strengthen this behavior. When you later face an aversive task, avoidance behavior tends to become more likely. In effect, the relations described by the ABC are assumed to be causal, but they describe causality in a complex model. Specifically,

- S^D^ alters the probability of some behavior because of a prior history of B-C relations under this S^D^
- B-C relations are causal in that C affects the *future probability* of B under a specific S^D^

As these relations are idiosyncratic, their nature must be determined in individual functional analyses. For procrastination, the basic ingredients are:

- S^D^: *Under what conditions do you procrastinate*? Most probably, there are several antecedent conditions that increase the likelihood of procrastination, and their identification is important to understand the consequences that, in turn, work as reinforcers.
- B-C relations: *When you procrastinate under such relations, what do you do, and what are the consequences*? Here, for each instance of procrastination, the specific behaviors must be specified, along with their typical consequence.

**Figure 1:**
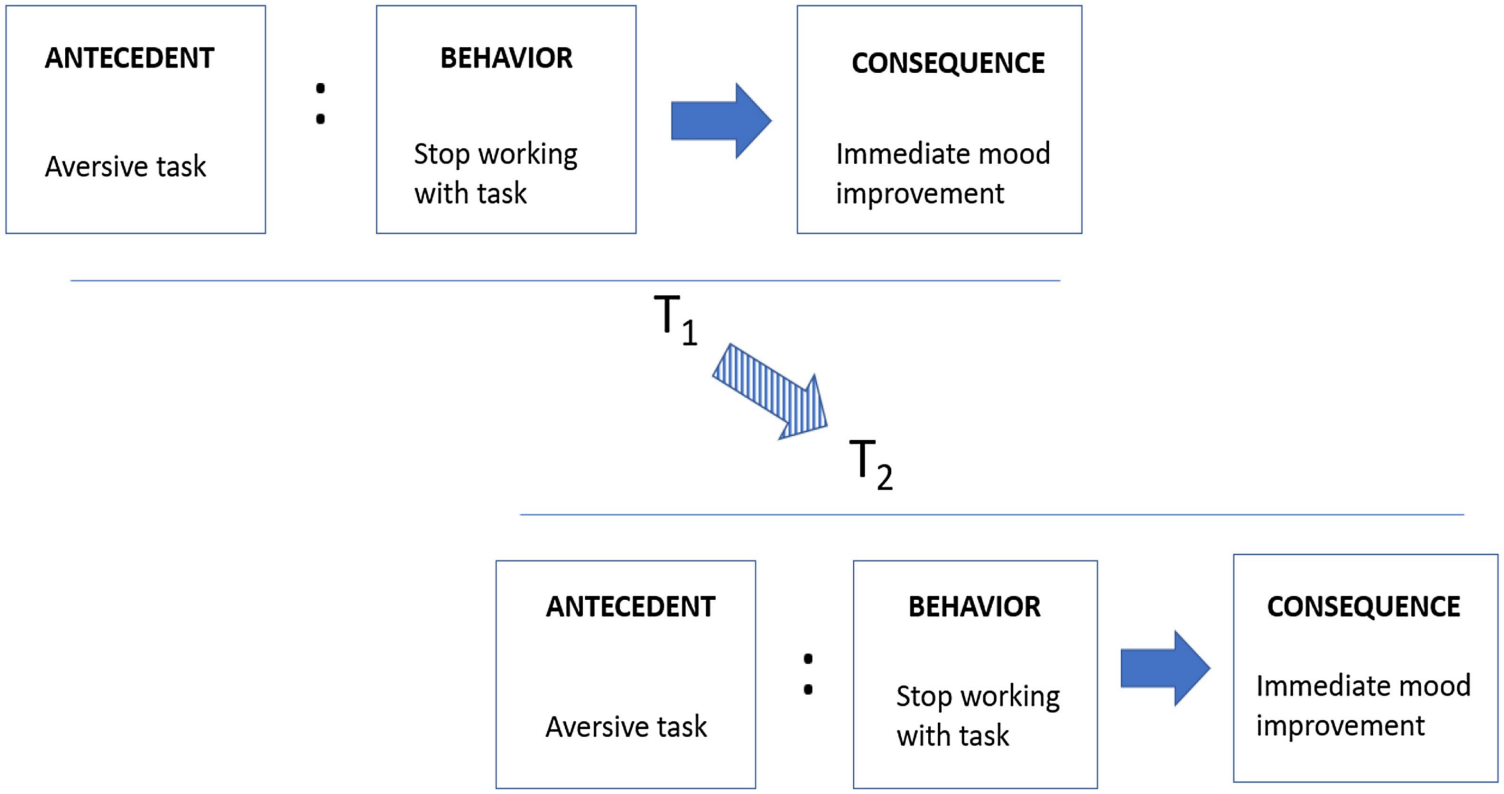
Schematic model of functional analysis, using negative reinforcement (escape) as an example. The antecedent (aversive task) sets the occasion for a procrastinatory behavior (stop task work), resulting in immediate mood improvement (consequence). Note that the Antecedent - Behavior relation is probabilistic (indicated by “:”), whereas the Behavior ➔ Consequence relation is causal. Note also that the negative reinforcement episode at T_1_ increases or maintains the probability that similar behaviors will be repeated under similar antecedents later (T_2_, and so on). Therefore, functional analysis is longitudinal and processual in nature, capable of capturing the dynamic effect of repeated reinforcement episodes.

Figure 1 summarizes the FA approach to understanding procrastination. It is beyond the scope of this paper to present the concepts and principles of FA. For a more comprehensive treatment of the principles of FA, consult Rachlin et al. (2018) or Pierce and Cheney (2017).

### How functional analysis may help understand procrastination

What could functional analysis (FA) add to the procrastination field? Although the answers to this general question must be assessed in individual analyses, research in the procrastination field provides several plausible paths to explore. As seen in Figure 1, *avoidance contingencies* may be a factor in academic procrastination when immediate mood improvement is achieved through escape and avoidance from academic work (e.g., Tice et al., 2001). Also, *appetitive contingencies* are likely when students shift from planned work to something more pleasurable, as in preference reversals (e.g., Steel et al., 2018). A third likely mechanism relates to *values* and *preferences*. If choices made during goal striving are associated with different values and preferences, attention to how such values and preferences relate to behavior is important (e.g., Grund and Fries, 2018). As academic tasks are often regarded as aversive (e.g., Blunt and Pychyl, 2000), this focus may be especially important in procrastination. Fourth, as the behavior that is procrastinated often will occur at a *lower frequency* than is needed for proper goal attainment, an understanding of how to increase the frequency of goal-relevant behavior is necessary. Here, behavioral activation (e.g., Veale, 2008; Kanter et al., 2010; Martell et al., 2022) presents well-documented techniques. In fact, research on behavioral activation for depression indicates that the use of simple strategies to increase behavioral frequency may also have a positive impact on the mood associated with the target behaviors. In academic procrastination, behavioral activation related to academic tasks should therefore be expected to be associated with reduced task aversiveness.

Next, how does this approach differ from traditional approaches to the procrastination problem? We briefly discuss five important dimensions.

1. Idiosyncratic. FA allows for an analysis of procrastination at the individual level (idiosyncratic approach), addressing specific characteristics and suggest appropriate measures to train goal-striving skills. As discussed, identification of individual profiles may be important both in the understanding of procrastination and in terms of intervention efforts (Steel and Klingsieck, 2016).
2. Focus on behavior.^2^ As procrastination is defined in terms of behavioral delay, a focus on behavior is a logical step (e.g., Svartdal et al., 2018). Because FA focuses on behavior in context, an analysis of procrastination in terms of the ABC model requires operationalizations of procrastinatory behaviors. As discussed, this may be a welcome initiative, as the definition of procrastination and its behavioral manifestations need refinement. By emphasizing behavioral function (rather than intentions or expectations) as a criterion for procrastination, FA may add precision to its measurement. Importantly, FA also allows for the specification of alternative behaviors with similar psychological functions but with less detrimental effects on goal attainment. In effect, alternatives to procrastinatory behaviors may be advised on an individual level.
3. *Mechanisms*. Although FA also focuses on the reflective system, an important focus of the ABC model is the impulsive system. As this system addresses automatic emotional and motivational processes as well as the learning history of the organism (Hofmann et al., 2009, p. 164), FA allows for unique access to important drivers of procrastinatory behaviors as well as effective means of reducing them. It is important to recognize that FA, contrary to common belief (e.g., Duckworth et al., 2018, p. 108), *does* include feelings and thoughts in the analysis of behavior. FA is inspired by Skinnerian behaviorism, and central publications by Skinner (e.g., Skinner, 1945, 1953,1974) make it clear that feelings and cognition are indeed central to FA. For example, Skinner’s 1945 paper outlined how people get to know their own feelings, an analysis that later inspired the development of self-perception theory (e.g., Bem, 1972). We emphasize this characteristic of FA because feelings and cognition probably are central to the understanding of procrastination. For example, if emotional regulation (or misregulation) is important in procrastination (e.g., Sirois and Pychyl, 2013), FA would focus on the relation between procrastinatory behavior (i.e., avoidance or escape behaviors) and the immediate “mood repair” that follow these behaviors. Clearly, behaviors that bring about immediate shifts from negative to positive moods are indeed at risk of being reinforced, indicating a potent mechanism that will increase or maintain the frequency of procrastinatory behaviors. Thus, a particularly strong feature of FA is its capability to tap into the dynamics of such relationships by means of an analysis proven valuable in basic and applied research over the last seven decades.
4. *Causal model*. FA is based on a causal model that is well capable of grasping the complexity of procrastination as it unfolds over time (e.g., Miltenberger et al., 1998). This is especially important in the procrastination field, as research has been dominated by correlational evidence (e.g., van Eerde, 2003a; Steel, 2007). The ABC model incorporates three basic causal mechanisms: a) *Reflexive S-R relations*, as seen when a stimulus reflexively elicits behavior in the individual; b) *Discriminative stimuli* (S^D^) that signal the availability of behavioral outcomes given a particular response; and c) *Behavior-consequence* (B-C) relations, contingencies between specific responses and desired outcomes. These relations unfold over time but in different ways. For example, B-C relations exercise their effect in “selecting” responses that are functional under specific conditions, and such selection is not observable momentarily but rather in later, similar situations. In general, behavioral sensitivity to the consequences of responding facilitates the individual’s adaptation to a particular environment. However, such sensitivity is finetuned to immediate behavioral consequences, as long-term consequences are discounted (e.g., Ainslie, 2012). As all these mechanisms describe how behavior is affected by them, it follows that changing these relations, for example, by changing a typical consequence of procrastinatory behaviors, will change behavior in predictable ways. Also, note that so-called motivational operations can affect these relations (Michael, 1982; Laraway et al., 2014). For example, whether you collect receipts when traveling may depend on you getting a refund (high reinforcer value) or not (low reinforcer value). Of particular relevance to procrastination are findings that indicate that framing of outcomes readily affects performance. For example, Ferrari and Tice (2000) demonstrated that a preparation time before an upcoming task defined as a “fun game” did not differ between procrastinators and non-procrastinators, whereas the same task framed as an “important evaluation of cognitive skills” resulted in procrastinators spending less time on practicing compared to non-procrastinators.
  As a cautionary note, FA may be used in different ways, from truly experimental to descriptive and interpretative, often called indirect or anecdotal (e.g., Iwata and Dozier, 2008). In the first case, experimental control is demonstrated by observing behavior in defined test conditions vs. experimental conditions, typically using N = 1 designs with repeated measures (e.g., Kazdin, 1998). In applied settings, experimental stringency may often be compromised. Here, functional analysis is used in a more descriptive or interpretative way, obtaining information about functional relations by use of interviews, checklists, rating scales, and questionnaires. Although such indirect or anecdotal approaches have their limitations, they are still recommended when the collection of direct-observation data is difficult (Herzinger and Campbell, 2007). Further, given that students can acquire the skills to understand and conduct FA sessions following very brief training (e.g., Iwata and Dozier, 2008), students may be able to self-report essential information needed for functional analyses and derived treatments.
5. *Appetitive and aversive control*. A final feature of the ABC model is that it can expertly analyze appetitive as well as aversive control conditions. As discussed, two dominating models of procrastination depict procrastinatory behaviors as the result of immediate reward conditions that “win” over long-time plans, as well as the operation of aversive conditions that create avoidance or escape procrastinatory behaviors that temporarily alleviate bad mood and stress. In the ABC model, both forms of control are well-known from basic as well as applied research, and the causal models incorporated in FA are well capable of analyzing the dynamics involved over time. In contrast, existing research in the procrastination field regards appetitive and aversive factors more as disruptors of planned work, with less focus on how such disruptions can strengthen future procrastinatory behaviors.

Table 1 summarizes the characteristics of FA in contrast to typical research in the procrastination field. It is important to note that the approach discussed in this paper is an integrative rather than an alternative approach. We incorporate important findings from procrastination research within the thinking of FA. In such efforts, it is essential to note that procrastination is defined in terms of a planned goal, such as an exam three months ahead. Some behaviors are functional in facilitating this goal, whereas other behaviors are not. Those that are not are termed “procrastinatory behaviors” if they occur at the expense of goal-relevant behaviors. To quote one of the best items to measure procrastination: “In preparation for some deadline, I often waste time by doing other things” (Item 1 from the General Procrastination Scale; Lay, 1986). Therefore, a key to understanding procrastination is to explain why such “irrational” and “maladaptive” behaviors occur during planned work. According to FA, the general answer is that dilatory behaviors occur because they tend to be “adaptive” or functional in the short time perspective but maladaptive in the longer time perspective. Thus, behaviors that are adaptive according to the longer-time perspective (e.g., preparation for an exam) are replaced by behaviors that are adaptive in the short-time perspective (e.g., mood repair).

**Table 1:** Differences between functional analysis and traditional procrastination research on five important dimensions.

	Functional analysis	Procrastination research
Approach	Idiosyncratic	Nomothetic
Definition	Behavioral function	Behavioral intention/expectation
Focus	Automatic, impulsive system; reflective, conscious system	Primarily reflective, conscious system
Analytical strategy	Causal, experimental	Correlational
Mechanism	Appetitive and aversive contingencies as drivers of the impulsive system	Appetitive and aversive events as disruptors of the reflective system

Given this perspective, an important role of *immediate controlling conditions* for behavior is indicated. In an early paper, Rachlin (1974) pointed out that self-control describes decisions about alternatives at different times, and the “breakdown” seen in self-control (or self-regulation) appears when the individual selects behaviors that adapt to short-term contingencies rather than to the long-term contingencies defined by the goal to be achieved. However, such decisions involve an element of preference. Thus, if the temporal perspective is removed, decisions about alternatives are reduced to preferences (Rachlin, 1974, p. 94). Accordingly, a functional perspective would examine behaviors during goal-striving, both procrastinatory and non-procrastinatory, in terms of the immediate consequences they generate and in terms of the perceived value of those consequences. As the temporal dimension is devaluated in procrastinators, it is likely that much of the procrastination problem boils down to a question of preferences. Thus, if a procrastinating individual had intended to read an important chapter today but instead did something more enjoyable, a devaluated time perspective for the reading activity implies that the decision is reduced to a choice between two activities present here and now: a) reading a chapter versus b) doing something enjoyable. This accentuates the perspective of FA to immediate drivers of procrastinatory behaviors. However, another implication is that long-term goals may be supported by immediate desired consequences of behaviors that facilitate goal-related behaviors, as in self-reinforcement (Bandura, 1976).

In the remainder of this paper, we explore in more detail the utility of functional analysis in the understanding and prevention/treatment of procrastination. As FA is simple to understand as well as to implement in preventive and curative efforts, knowledge of basic principles might help the student as well as teachers and counselors to understand the ABCs of procrastination and thereby become capable of identifying and changing controlling conditions. FA might be seen as a tool for “awareness-raising” or insight into factors contributing to the individual’s procrastination. In the next section, we explore the procrastination literature related to the Antecedent conditions for procrastination, procrastinatory Behaviors, as well as typical Consequences of procrastinatory episodes.

## Factors associated with procrastination and how they align with functional analysis

### Antecedent conditions

In functional analyses, antecedent conditions (A or S^D^) represent signals or conditions for specific behaviors, as well as signals for typical consequences associated with those behaviors. We discuss three examples of classes of antecedent conditions that all serve this function. The classes are well-documented and may be common to many procrastinators.

#### Situational temptations and distractions

A potent antecedent for procrastination is temptations present during goal-directed work. For example, if friends meet socially when you are working on an assignment, it may take extra effort to continue. Continued work under such conditions is a typical example of self-control (e.g., Tice and Baumeister, 1997) – working with a distant desired outcome in mind rather than giving in for the pleasure of socializing. Exercising self-control under tempting conditions is itself aversive (Tice and Bratslavsky, 2000; Tice et al., 2001; Sirois and Pychyl, 2013), which may make situational temptations even more tempting. Research has documented situational temptations to be strongly correlated with procrastination. For example, “emotional distractibility” (Heckhausen and Dweck, 1998) addresses difficulties in resisting temptations (example item “I let myself get distracted by more pleasant things”), demonstrating a correlation of *r* = 0.72 with procrastination (Steel et al., 2018). Situational temptations and distractions represent signals for preference reversals during goal striving. In the ABC language, temptations and distractions are typical antecedent conditions that signal the availability of immediate desired outcomes, given procrastinatory behaviors.

#### Task aversiveness

One of the best-established findings in the procrastination literature is that task aversiveness reliably predicts procrastination (Schraw et al., 2007; Grunschel et al., 2013; Klingsieck et al., 2013). Steel (2007, p. 75) concluded in his meta-analysis that the correlation between procrastination and task aversiveness is relatively strong, *r* = 0.40. This correlational evidence is strengthened by experimental evidence (Senecal et al., 1997). Blunt and Pychyl (2000) examined 30 potential dimensions related to task aversiveness and found boredom, frustration, and task resentment to be the dimensions most strongly associated with perceived aversiveness over various stages of task execution. Other factors may instigate aversiveness strategies as well. Long deadlines may make the individual infer that the task at hand is difficult, which may prompt unnecessary delay (Zhu et al., 2019). Physical effort is itself inherently aversive (Eisenberger, 1992), and individuals are more likely to procrastinate on effort-demanding tasks (Milgram et al., 1988). Lack of interest may also be associated with task aversiveness (Song et al., 2019). Aversiveness has also been conceptualized as an overall avoidance of deadlines present in goal-directed behaviors (van Eerde, 2003b). Ferrari et al. (1995) linked avoidant behaviors to the protection of self-presentational image, procrastinators being overly concerned with avoiding situations that might reveal an adverse negative image.

As in the case of temptations/distractions, task aversiveness is a signal for preference reversals during goal striving, motivated by the expectation that procrastinatory behaviors bring about an immediate reduction in negative feelings.

#### Lack of energy and tiredness

Task aversiveness may be regarded as a relatively static property of tasks. However, task aversiveness is often related to fluctuating factors, some depending on individual characteristics. For example, lack of energy and tiredness may make tasks appear aversive or tiresome to engage in. Lack of energy is reliably and strongly associated with procrastination. Gröpel and Steel (2008) found a strong correlation of *r* = 0.60 between procrastination and energy level with a large, diverse sample of 9,351 participants, a finding later repeated by Steel et al. (2018). Low energy may be a likely instigator of procrastination because work becomes painful or more difficult to initiate when energy is low (Burka and Yuen, 1983; Baumeister et al., 1994, 2000). Low energy may also weaken self-control (Baumeister, 2002), making the individual more susceptible to situational temptations and distractions. In a large-scale Norwegian study (*N* = 50.000), students reported lack of energy and tiredness as the dominating health problem (Knapstad et al., 2021). Analyzing this data set, Svartdal and Gamst-Klaussen (unpublished) found that sleep problems accounted for a substantial part of the variance associated with lack of energy/tiredness. Other research (e.g., Kroese et al., 2014) further links sleep problems with procrastination in a direct way, as “bedtime procrastination” may itself be an important source of sleep problems.

Hence, the subjective experience of lack of energy may be a strong antecedent signaling that avoidant behavior pays off in terms of temporary relief and mood repair.

#### Factors interacting with antecedents

Antecedent conditions work in conjunction with other factors that may prompt the individual to delay through other mechanisms. For example, in the case of long deadlines, the desired outcome is temporally distant. Temporal distance to the desired outcome is a well-documented factor involved in procrastination (*cf.* TMT; Steel and König, 2006). Also, experimental studies indicate temporal elasticity in performing the same task. Thus, manipulation of the time available for tasks invites individuals, in accordance with Parkinson’s law, to fill the time available for completion (e.g., Brannon et al., 1999). Further, Huang et al. (2021) demonstrated that the way students are prompted to action in assignment completions may be important. Just reminding students about a deadline had a counterproductive effect, whereas descriptive norms (i.e., communicating peer assignment completion rates) had a positive effect on submission rates. Thus, in identifying antecedent conditions, care should be taken to identify antecedents that set the occasion for detrimental B-C contingencies in contrast to other forms of information and conditions that work through other mechanisms.

Another important set of factors that must be considered is individual difference variables. For example, an individual high in conscientiousness would probably be immune to situational temptations during goal striving. For this reason, analyses of antecedent stimuli should also address relevant individual difference variables in a systematic way. Importantly, some difference variables demonstrate very low or no correlations to procrastination, such as age, gender, intellectual capability, fear of failure, and perfectionism (van Eerde, 2003a; Steel, 2007). On the other hand, procrastination correlates moderately to highly with conscientiousness (*r* = −0.63), self-efficacy (*r* = −0.44), and self-handicapping (*r* = 0.46). Such correlational evidence should guide the analysis of antecedent conditions. There may be a core in all forms of procrastination, but the way procrastination is expressed may depend on individual characteristics (see Steel and Klingsieck, 2016). Individual difference variables are relatively easy to assess using standardized scales and should be included in tailoring interventions.

#### Summary and evaluation

We examined three classes of well-documented correlates of procrastination. As discussed, the function of antecedents is not only to increase the probability of procrastinatory behaviors. According to FA, antecedents may also have an important function of signaling typical consequences of procrastinatory behaviors under these conditions. These consequences, in turn, have the function of reinforcing procrastinatory behaviors. Such antecedent ➔ behavior ➔ consequence relations will, if left unchanged, maintain or strengthen the procrastination habit. Furthermore, because these relations are learned, they can be unlearned or relearned. For example, when the B-C relation is subjected to extinction conditions or is followed by alternative, non-procrastinatory behavior, the antecedent conditions signal that the B-C relation is weakened. Similarly, Eisenberger (1992) demonstrated that effort aversiveness may be reduced by pairing high effort with reward (learned industriousness). If tasks are perceived as boring or difficult (i.e., aversive) because academic skills are low, training of relevant skills may make such tasks more interesting and less difficult (e.g., Svartdal et al., 2021). Lack of energy/tiredness may be approached more directly with intervention efforts to improve sleep hygiene (e.g., Suh et al., 2021). Finally, antecedent conditions interact with factors that work through other mechanisms, and antecedent conditions affect individuals differently.

### Behaviors involved in procrastination

The standard criterion for procrastinatory delay behaviors is the subjective expectation of being worse off because one opts to delay (e.g., Steel, 2007). From the perspective of FA, such expectations of outcomes are secondary. According to FA, the primary issue is *behavioral function*: When procrastinating, under what circumstances do procrastinatory behaviors occur, and what have been the immediate consequences of these behaviors in the past?

Research has provided a variety of examples of delays involved in procrastination. For a functional approach, a brief overview of common behavioral operationalizations of procrastination is necessary. Note, however, that many studies do not focus on behavior but rely on scale scores measuring self-reported procrastination. Such scales may measure general (trait, dispositional) procrastination, state procrastination (the reported occurrence of procrastinatory behavior), procrastination in a specific context (e.g., academic), and others (see Svartdal and Steel, 2017).

#### Task completion

When researchers have addressed the *behavioral delay* occurring in procrastination, they have almost exclusively operationalized this notion to mean goal attainment or task completion. Examples include the postmark on participants’ envelopes when submitting a survey (Lay, 1986), the completion time of a research study (Solomon and Rothblum, 1984), turning in papers “late” or “early” (Tice and Baumeister, 1997), and the number of days participants took in returning a folder (Ferrari, 1992). In all these cases, delays may be influenced by prioritization of tasks, advice from others to delay, and other legitimate reasons that may reduce the validity of these behavioral measures. Tuckman (1991) seemed to specify a more valid measure of behavioral procrastination – voluntary homework assignments, where students could write and submit written material to gain extra course credits. Clearly, fewer assignments delivered would be indicative of behavioral procrastination, as both delay and a disadvantage were involved. Tuckman demonstrated a negative correlation between this measure and procrastination scale scores, *r* = −0.54, allowing him to conclude that “students are well aware of their own tendencies and can report them with great accuracy” (p. 9). In sum, if task completion implies a disadvantage to the individual, delayed completion is likely to be indicative of procrastination. However, as this measure includes non-procrastinatory delays, it should be handled with caution.

#### Behavioral onset

Researchers have repeatedly pointed out that onset delay, or intention-action gap (Steel et al., 2001; Steel, 2010), constitutes a core problem in procrastination. Svartdal et al. (2020, Figures 3, 5) asked students to rate the perceived difficulty associated with different action phases and found that getting started (onset) was reported to be especially troublesome, particularly for high procrastinators. Scales measuring procrastination include items to address the intention-action gap (e.g., item 2 in the DPS, “Even after I make a decision I delay acting upon it”). Delayed action on decisions often means that the individual prioritizes task-irrelevant activities (e.g., GPS item 1 “In preparation for some deadline, I often waste time by doing other things”; Lay, 1986).

It should be noted that late behavioral onset does not imply that “the faster the onset, the better.” Hasty and impulsive responding to action possibility, especially complex ones, may not be optimal. For example, immediate responses to e-mails increase the likelihood of making errors. Thus, *precrastination* (e.g., Rosenbaum et al., 2014; Wasserman, 2019) may be the opposite of procrastination but not necessarily an adaptive strategy either. Most new tasks must be embedded in ongoing projects that are ranged according to priority. Hence, although delay, as measured from the start of the response window to onset, may be an indicator of procrastination (e.g., Miyake and Kane, 2022), this measure includes delays that result from planning and prioritization and cannot be regarded as a precise measure.

#### Sustained goal work

As most tasks require sustained effort over time, successful goal attainment requires continued work over days or weeks once the individual has started. In this phase, procrastination manifests itself as impulsive shifts from the ongoing activity to other tempting activities. Note that although the distinction between onset delay and delays during goal striving seems to be intuitively meaningful, “onset delay” (the intention-action gap) has been taken to refer to getting started the first time (“finally, I started work on my thesis”). But “onset” also may refer to reengagements in more comprehensive tasks that require several onsets or reengagements (e.g., start work again on my thesis after breaks and pauses).

#### Experience sampling

In recent years, experience sampling (e.g., Csikszentmihalyi and Larson, 2014) has been used to obtain more detailed behavioral measures about behaviors involved in procrastination (e.g., Pychyl et al., 2000). In this procedure, respondents are prompted to report on ongoing activities, thus identifying occurrences of procrastinatory behaviors (e.g., watching TV) versus behaviors functional in goal attainment (e.g., studying). As will be discussed, this method of assessing procrastination shares some similarities with the approach suggested by FA, but it lacks the broader focus of FA in assessing control conditions.

#### Behavioral dimensions to address in FA

Given these considerations, it seems quite clear that FA would recommend alternative operationalizations, as well as alternative ways to handle them. We briefly discuss four operationalizations.

1. *Response competition*. Behavioral procrastination implies that the appropriate behavior (e.g., reading a chapter) is replaced by some other behavior (e.g., having a coffee with a friend), particularly so under conditions that are intended for goal-directed work. To assess the nature and frequency of such forms of behavioral delay, individual activity monitoring is indicated. Activity monitoring and subsequent activity scheduling are the first steps in behavioral activation (Kanter et al., 2010). For example, in the monitoring of depression (Veale, 2008), daily activities are initially monitored using a detailed coding system that, hour by hour during the day, specifies the concrete activities done as well as the mood associated with the activity. In the case of academic procrastination, a similar approach may be used (e.g., Suh et al., 2021; Martell et al., 2022). Here, the occurrence of non-functional and functional behaviors in study settings is recorded along with the typical consequences (including emotional changes) associated with these behaviors. In this way, the occurrence of functional versus dysfunctional behaviors and their typical antecedents and consequences are recorded as chains, also described as chain analysis in CBT (Rizvi and Ritschel, 2014). It is likely that the results from activity monitoring will correlate highly with scale scores measuring (academic) procrastination, but the obvious advantage of activity monitoring is that procrastinatory (and non-procrastinatory) behaviors are specified concretely and in context, making subsequent intervention in terms of activity scheduling possible.
  Activity scheduling then specifies activities the individual is recommended to perform more often, as well as activities the induvial is advised to perform more rarely. In the treatment of depression, such activities are scheduled with the aim of improving mood. In procrastination, mood may be a secondary aim, as the primary goal is to increase the proportion of goal-relevant activities relative to the proportion of goal-irrelevant activities. Here, activity scheduling should provide relatively detailed information on the triggers that typically instigate procrastinatory behaviors, as well as the typical consequences these behaviors produced under those conditions. Such information provides specific insight into the control conditions for different forms of procrastination for a given individual and on conditions that may be changed to reduce procrastination.
2. *Delay of preparatory behaviors*. Procrastination often involves preparatory behaviors, i.e., behaviors that are necessary or beneficial for later behaviors to be executed efficiently (Sheeran and Webb, 2016). Within the framework of self-regulation, preparatory behaviors work to facilitate the implementation of planned behavior (e.g., Barz et al., 2016; St Quinton and Brunton, 2017). Preparatory behaviors may facilitate a target goal regardless of their motivational or volitional origin. For example, following advice from a teacher to read a chapter before a lecture is beneficial to the target behavior, even if it is unplanned by the student. Similarly, organizational measures that require students to complete parts of complex tasks in pre-defined temporal succession (e.g., Baars et al., 2021) may facilitate target goal attainment.
  Although such preparatory behaviors are rarely examined in procrastination research, it is well known that procrastinators tend to come unprepared for classes and do last-minute preparations for classes and exams (e.g., Solomon and Rothblum, 1984; Milgram and Naaman, 1996). Failure to perform preparatory behaviors, or delays in them, may negatively affect subsequent target behaviors and should thus be regarded as potentially important contributors to the procrastination problem (see Svartdal et al., 2018). As preparatory behaviors may be less available to monitoring compared to tempting activities that compete in the current situation, the occurrence of this form of delay is probably difficult to access in self-report instruments and probably also in measures of behavioral procrastination. For this reason, a simple way to alleviate the lack of preparatory behaviors in the academic context is to implement structural and organizational measures that require students to complete parts of complex tasks in pre-defined temporal succession (e.g., Svartdal et al., 2020; Baars et al., 2021). Alternatively, preparatory behaviors may be monitored and changed as part of activity monitoring and scheduling.
3. *Proactive self-regulation*. As discussed, recent developments in research on self-regulation indicate that proactive self-regulation is important in individuals demonstrating high trait self-control (for an overview, see Inzlicht et al., 2020). Interestingly, behavior modification, which is based on functional analysis, has long emphasized the utility of stimulus control in the management of goal-directed behavior (e.g., Mahoney and Thoresen, 1972), for example, in terms of strategies to avoid tempting future situations or by removing temptations from the current situation. As procrastinators are low in trait self-control (e.g., Zhao et al., 2021), present-oriented, and less apt to simulate future situations concretely (e.g., Sirois and Pychyl, 2013; Rebetez et al., 2016), it follows that procrastinators may demonstrate less proactive self-control compared to non-procrastinators. By using proactive self-regulatory techniques (e.g., Sklar et al., 2017), individuals may reduce the likelihood of being exposed to situations that require effortful self-regulation. In general, proactive self-regulatory techniques may *manipulate the situation* (e.g., changing the situation to prevent temptations from occurring) or *reduce the effect of some temptations* (e.g., by not attending to them). Creating rules for oneself in *pre-commitment* is also an effective strategy to enhance self-control (e.g., Ariely and Wertenbroch, 2002), as is *implementation intentions* (Gollwitzer and Sheeran, 2006). As for lack of preparatory behaviors, lack of proactive self-control may be difficult to measure behaviorally. However, a recent self-repost scale (the Self-Control Strategies Scale; Katzir et al., 2021) includes a subscale that addresses the use of specific proactive self-control strategies. Further, as for preparatory behaviors, proactive strategies may be implemented in conjunction with activity monitoring and activity scheduling.
4. *Self-handicapping*. Self-handicapping is a form of active self-sabotaging that people perform to establish specific external events as excuses if subsequent performance fails. Thus, rather than forgetting or postponing preparatory behaviors, self-handicapping implies a tendency to engage in strategically delayed behaviors that protect self-esteem (e.g., Strube, 1986; Schwinger et al., 2021). For example, staying up late the night before an important exam may later be used as an excuse if the exam results are not as positive as expected. van Eerde (2003a) found in her meta-analysis a relatively strong correlation between self-handicapping and procrastination, *r* = 0.48. As self-handicapping is rooted in concrete behavioral choices, the occurrence of such behaviors may be assessed in activity monitoring and changed through activity scheduling.

#### Interacting factors

Even though consequences tend to affect behavior, it is important to recognize that other factors may affect their role in behavioral control. For example, low outcome expectations for the target behavior (e.g., in the form of low self-efficacy) may strengthen the effect of immediately tempting alternative behaviors. Similarly, the ability to concretely represent desired outcomes of the focal task (e.g., in future episodic thinking) may weaken the effect of immediately available tempting alternatives.

#### Summary

This brief discussion indicates that FA approaches the operationalization of behavioral procrastination in ways different from traditional approaches in two important ways. First, procrastinatory behaviors are analyzed in terms of their function. Function is assessed individually in relation to immediate consequences, given specific antecedent conditions. This way of operationalizing procrastinatory behaviors allows for a more precise identification of procrastination compared both to the self-report approach and to prior attempts to define behavioral procrastination. Second, FA addresses procrastinatory behaviors in a more comprehensive way compared to most other approaches. Thus, currently available behavioral options, procrastinatory as well as non-procrastinatory, are the main focus. In addition, behaviors that precede them (e.g., preparatory behaviors; proactive behaviors) may be included in the analysis. Importantly, by focusing on behaviors that temporally precede the targeted procrastinatory behaviors, the individual may better realize how prior behaviors have consequences here and now and how they may be changed. For assessment as well as change, a well-documented and clinically validated approach to behavioral change, activity scheduling (Kanter et al., 2010), is available. Table 2 summarizes and exemplifies the four behavioral forms of procrastination discussed.

**Table 2:** Procrastinatory behaviors, given “working with an assignment” as the activity planned.

Behavior	Example
*Disadvantageous behavioral delay*, especially those forms with late onset of task behavior (intention-action gap)	Delay starting work with an assignment when there is no good reason for the delay
*Preference for competing behaviors* with immediate reward contingencies	Quit or delay working on an assignment to do something more pleasurable
*Delay or omission of preparatory behaviors*	Did not prepare for a planned group meeting about an assignment
*Self-handicapping*	Strategically did not prepare for a planned group meeting about an assignment

## Consequences of procrastination: Immediate and delayed consequences

An important motivation for the functional analysis of procrastinatory behavior is the assumption that the consequences of procrastinatory behaviors are important in controlling this habit. As discussed, one of the dominating models of procrastination is an emotion-regulating model that regards procrastination as a self-regulatory failure, with short-term mood repair and emotion regulation as the main ingredients (Sirois and Pychyl, 2013). From the perspective of functional analysis, this model can be viewed as an excellent example of negative reinforcement.

### Removal of aversive states: Negative reinforcement

Negative reinforcement is described by a three-term contingency (ABC) where aversive antecedents (e.g., boring tasks) set the occasion for avoidance or escape behaviors. Behaviors that immediately remove, postpone, reduce, or in other ways lessen the aversiveness are reinforced. In an early paper, Tice and Bratslavsky, (2000) pointed out that when we work with aversive tasks, we “give in to feel good” simply by escaping or avoiding the task. Later, Sirois and colleagues published important work to bolster the mood repair and emotion regulation model of procrastination (e.g., Sirois and Pychyl, 2013; Sirois and Kitner, 2015). A central feature of this model is that mood repair is immediate, given a procrastinatory behavior (e.g., Tice et al., 2001). In terms of FA, the short delay between procrastinatory behaviors and desired consequences represents a powerful B ➔ C contingency, meaning that reinforcement of procrastinatory behaviors is likely. In effect, such behaviors will tend to occur with a high probability in the future. Because immediate consequences in negative reinforcement (e.g., mood repair) have a stronger effect compared to delayed detrimental consequences of abandoning planned work, procrastinatory behavior is strengthened without an effective corrective mechanism.

Negative reinforcement includes two variants. In *escape*, behavior removes the aversive stimulus from the situation. In *avoidance*, behavior prevents the occurrence of the disliked stimulus altogether. Both forms have been well documented in animal and human research, with avoidance learning being considered especially important (e.g., Krypotos et al., 2015). First, the condition that is avoided is idiosyncratic, meaning that any subjective feelings of stress, dislike, fear, or similar suffice as motivators for avoidance behavior. Second, the behavior that prevents negative feelings will be effective in strengthening the avoidance behavior, is based on subjective judgment – the belief that the B-C relation works is sufficient. Third, avoidance behavior may continue almost indefinitely, even in the absence of a real B-C contingency. For example, if you dislike (or fear) presenting at seminars, simply avoiding seminars will remove the threat. Such avoidance behavior will be “effective” even when the seminar leader (unknown to you) has announced that student presentations will not be required or encouraged.

An unfortunate side-effect of negative reinforcement contingencies is that focus is on avoiding or removing aversive states, with the potential of narrowing the individual’s behavior repertoire (Ferster, 1973). Thus, avoidance contingencies tend to foster lower behavioral variability, strengthen negatively motivated behavior, and ultimately dispose the individual of passivity and depression-like thoughts and feelings (e.g., Veale, 2008).

### Added positive events: Positive reinforcement

Whereas negative reinforcement acts by removing an aversive state dependent on (procrastinatory) behavior, positive reinforcement has its effect when an event is added to the situation dependent on behavior. As planned behavior is under the control of delayed consequences, other activities with desired consequences available now or soon are likely to compete and often win over the planned behavior. Preference reversal, the procrastinating individual’s willingness to make plans only to reverse plans before goal accomplishment (Steel et al., 2018), is a predictable outcome of such short-versus long-time reinforcement contingencies.

Under a functional analysis, two important variables contribute to such preference reversals. First, situational temptations are potent reinforcers that will strengthen behaviors that produce them. In individuals who feel attracted to impulsive diversions from plans and/or are easily distractable, situational temptations may become close to irresistible. In fact, the correlation between procrastination and distractibility is high, *r* = 0.64–0.72 (Steel et al., 2018), and susceptibility to temptation (as measured by the STS; Steel, 2010) correlates also highly with procrastination, *r* = 0.59–0.71 (e.g., Svartdal et al., 2016; Rozental et al., 2022). These findings indicate that positive reinforcement contingencies for procrastinatory behavior in the form of “doing something else than I had decided to do” may strengthen procrastination, and especially so in individuals scoring high on scales measuring impulsiveness, susceptibility to temptation, or distractibility. Second, if the goal-directed activity is to some extent aversive, attractive situational stimuli become even more tempting. As discussed, exercising self-control is itself associated with negative feelings, implying that negative feelings may appear from aversive tasks as well as from one’s own struggle in maintaining goal striving.

### Sub-classes of behavior-consequence relations

Response competition implies that behavior with immediate desired consequences tends to dominate over behaviors with long-term consequences. In effect, impulsiveness (i.e., impulsive deviations from plans) may be reinforced (see Svartdal et al., 2018). Impulsiveness correlates highly with procrastination (Steel, 2007; see also Gustavsson et al., 2014), but it is likely that impulsive diversions are not only a function of an impulsiveness personality trait but also result from a previous reinforcement history. In terms of functional analysis, impulsiveness may occur both under positive and negative reinforcement contingencies. In the first case, reinforcement by situational temptations (alternatives to planned goal striving) is a well-known mechanism; in the second, willingness to tolerate an aversive state for a long-term goal (i.e., resilience to tolerate and accept aversive emotions; Berking and Whitley, 2014) is undermined when the individual experiences swift removal of aversiveness just by quitting planned work. Note that in both cases, the immediacy of the reinforcing events is likely to be powerful in fostering impulsiveness.

### Summary

We have discussed the two main mechanisms for how the consequences of procrastinatory behaviors work to strengthen such behaviors (see Table 3). “Strengthen” here simply means that consequences of a procrastinatory behavior on one occasion tend to make such behaviors likely under similar conditions later. Functional analysis of procrastination must identify the behavior-consequence relations that seem to be dominating for a given individual and then suggest alternative behaviors that can reduce procrastination. Behavioral consequences may possibly strengthen other forms of behavior, as impulsiveness.

**Table 3:** Procrastinatory behaviors and consequences, given “working with an assignment” as the activity planned.

Behavioral consequence	Example
*Negative reinforcement*: Immediate removal of aversive state following procrastinatory behavior	Working with assignments is aversive, just quitting produces immediate mood repair
*Positive reinforcement*: Immediate presentation of a desired state following procrastinatory behavior	Quit working on an assignment and instead do something more pleasurable

## A functional analysis of academic procrastination: Summing up

### Important contributions from FA

As is apparent from the preceding discussion, the fit between analysis in terms of FA and traditional approaches is good. However, when it comes to the understanding of procrastinatory behavior, differences are obvious. Another difference relates to analytical strategy.

#### Definition and criteria

Central to a research field is that important concepts are clearly defined and well operationalized. Although the procrastination construct is reasonably well defined (i.e., behavioral delays that are detrimental to the individual), the criteria needed for separating procrastination from non-procrastinatory delays are not. In current definitions, the subjective intention when deciding to delay planned tasks is regarded as crucial (e.g., Steel, 2007; Klingsieck, 2013). However, as such intentions are difficult to measure and are also rarely measured, the criteria for identification of procrastinatory behaviors need refinement. Problems associated with this subjective criterion (e.g., overoptimistic beliefs, harsh self-evaluation, strategic thinking that did not work, and others; see Svartdal and Steel, 2017; Svartdal and Nemtcan, 2022) reinforce this conclusion.

The ABC model takes a very different view, not on the definition itself but on the criteria needed to identify procrastinatory behaviors. Rather than focusing on intention, FA focuses on function: How are specific behaviors involved in the delay of planned activities, and what are their controlling conditions? Assessment of function must be done at the individual level, as the very same behavior (e.g., skip reading and do something else instead) may be controlled by different contingencies in different individuals (e.g., skip reading because the book is aversive or boring, or skip reading to have a coffee with a friend). As discussed, the methodology to identify behavioral function at the individual level is available (e.g., Martell et al., 2022), and it is highly probable that the resulting individual profiles will be useful not only in identifying procrastinatory behaviors but also in identifying control conditions that in turn may be used to prevent or reduce procrastination.

Another characteristic of FA is that procrastination is defined and operationalized behaviorally. This represents a significant step forward, as most procrastination research has used self-report scales despite the fact that procrastination is defined in terms of behavior. Also, as discussed, in cases where researchers have attempted to use behavioral measures, those measures have often been of questionable quality. The behavioral measure suggested by FA analyzes individual behavior in context using well-established procedures. By using activity monitoring and chain analysis, behaviors that demonstrably hamper planned goal attainment, as well as behaviors that are functional in goal attainment, are assessed. Activity monitoring and chain analysis provide a detailed account of possible control conditions for procrastinatory behaviors (their “function”) and hence of possible ways subsequent activity schedules may be administered.

Previous studies on (academic) procrastination have attempted to identify different typologies based on scale scores (e.g., Solomon and Rothblum, 1984; Milgram and Naaman, 1996; Schouwenburg, 2004; Steel and Klingsieck, 2016). The ABC approach takes typology to another level, making it possible to identify profiles of control conditions for procrastination at the individual level. However, in contrast to common approaches, the purpose of such analyses is not to formulate general knowledge of procrastination but to identify profiles that may be useful for prevention and reduction of procrastination.

#### Model and analytical strategy

Procrastination is a dynamic phenomenon, capable of being a cause, an outcome, and a correlate. As discussed, the general understanding of procrastination as a form of breakdown in self-regulation (e.g., Steel, 2007) is not very informative, given the complexity of this construct (e.g., Inzlicht et al., 2020). Furthermore, the procrastination field has been dominated by correlational studies, although studies in recent years have started to apply advanced statistical techniques, often in longitudinal designs (e.g., Steel and Svartdal, 2022). Experimental studies are rare.

The ABC model represents a fresh approach. First, functional analyses may address drivers of the automatic impulsive system (e.g., Hofmann et al., 2009; Heatherton and Wagner, 2011). Whereas traditional approaches to the drivers of procrastinatory behaviors regard them as a relatively static function of personality characteristics (e.g., impulsiveness, distractibility), FA assumes that such drivers are capable of being strengthened or weakened dependent on experience. The well-established findings that procrastination may result from dysfunctional emotional regulation (e.g., Sirois and Pychyl, 2013; Sirois and Kitner, 2015) is a case in point. The immediate short-term mood repair appearing as a consequence of procrastinatory behaviors indicates that such behaviors are “adaptive” in the short-term perspective – hence they are reinforced. Over time, this will then strengthen the tendency to procrastinate. This dynamic effect of the consequences of procrastinating has largely been overlooked in the procrastination literature.

As the ABC model does not distinguish between automatic, impulsive behaviors and behaviors related to the reflective system, another strength of the ABC model is that procrastinatory behaviors related to the latter system may also be subjected to functional analysis. This is particularly relevant in interventions to increase the frequency of goal-relevant behaviors. For example, using activity scheduling (e.g., Kanter et al., 2010), the occurrence of non-procrastinatory behaviors may be increased. Such interventions may address most forms of procrastination, both those representing short-sighted choices between activities here and now (e.g., choosing to watch TV rather than reading) as well as activities that facilitate or hamper later activities (e.g., reading a recommended chapter before a lecture). By focusing directly on behavior, FA has a very concrete approach to the procrastination problem.

#### Interventions

Building on the promising results from meta-analyses demonstrating that CBT is an effective form of intervention against procrastination (Rozental et al., 2018; van Eerde and Klingsieck, 2018), a logical step forward is to incorporate FA. First, as suggested by Steel and Klingsieck (2016), interventions should be adapted to individual characteristics. The very nature of FA is to adapt intervention efforts to individual profiles. Second, as illustrated throughout this paper, important insights from procrastination research may be integrated into the thinking of FA. For example, in individual assessments of procrastination, standard personality tests addressing procrastination-relevant dimensions (e.g., extraversion, impulsiveness, neuroticism) may indicate important factors to consider. Third, the relative simplicity of the FA approach to assessment and interventions makes this method particularly interesting in the prevention and reduction of academic procrastination. No clinical expertise is required, but ability and willingness to address personal procrastination in a systematic way are needed. Behavioral chain analysis (ABC-analysis) is well-known in dialectical behavior therapy and CBT (Tirch et al., 2016). Finally, the ABC model has proved viable in interventions for problems related to procrastination (Cuijpers et al., 2007; Dimidjian et al., 2014; Simmonds-Buckley et al., 2019; Stein et al., 2020), and detailed accounts of assessment and intervention procedures are available (Suh et al., 2021; Martell et al., 2022).

Clearly, interventions may also apply other strategies to supplement FA. As an important mechanism involved in procrastination seems to be related to dysfunctional emotional regulation, especially of negative emotions (e.g., Tice and Baumeister, 1997; Tice and Bratslavsky, 2000; Sirois and Pychyl, 2013, 2016; Pollack and Herres, 2020), interventions that focus on emotion regulation skills are indicated. Indeed, using the Adaptive Coping with Emotions Model (ACE Model; Berking and Whitley, 2014), Schuenemann et al. (2022) demonstrated that an intervention to train emotion regulation skills can reduce procrastination. Inspecting the ACE Model, effective emotion regulation addresses several aspects related to the antecedent part of the ABC model, such as *emotion perception* (i.e., to be aware and correctly identify and label emotions), identification of *causes and maintaining conditions* for emotions, the ability to *tolerate negative emotions* when necessary (i.e., emotional resilience), being able to a*pproach and confront situations that are likely to trigger negative emotions,* and being able to *provide self-support in distressing situations*.

### Limitations of FA

A fundamental barrier to adopting FA in the analysis of psychological topics is a heritage from the cognitive revolution. FA is rooted in the behavioristic tradition, and the view that psychology “could not participate in the cognitive revolution until it had freed itself from behaviorism, thus restoring cognition to scientific respectability” (Miller, 2003, p. 141) was widespread. As a result, decades of studies on the psychology of learning seemed to suffer in the process (e.g., Reber, 2004). However, as pointed out by several authors (e.g., Leahey, 2002; O'Donohue et al., 2003), the nature of the cognitive revolution was exaggerated by many. We argue that scientific contributions must be evaluated in terms of their value, both in scientific and practical terms. As argued repeatedly in this paper, that value seems to be unquestionable in dealing with the procrastination problem.

The question then arises as to whether the FA model is sufficiently compatible with a standard approach to procrastination. As argued throughout this paper, the answer is yes. Apart from differences in the operationalization and analysis of procrastinatory behaviors, we see no substantial barrier to integration. Specifically, an analysis in terms of FA will clearly benefit from the vast database of knowledge in the procrastination field. Furtheer, the current problems seen in the procrastination field regarding definitional criteria, theoretical understanding, and interventions all indicate that new impulses are welcome.

Another important issue is whether human behavior at all is affected by learning contingencies in the sense that is assumed in FA. For example, is human behavior at all sensitive to behavioral consequences? And if yes, do we need analysis in terms of conditioning? The answer to the first question is yes. In answering the second question, one view is that conditioning (learning of instrumental response-consequence relations) depends on cognition (the reflexive system; e.g., Shanks, 1993). Others recognize that behavior may be changed in ways that escape conscious apprehension (e.g., Kirsch et al., 2004). In the present context, it is an important premise that human behavior is sensitive to consequences, regardless of whether this sensitivity is apprehended consciously or not. Importantly, we should recognize that at least some forms of change by consequences are not easily apprehended by the individual. For example, change by consequences may be differentially influenced by antecedents, such as when an individual tends to procrastinate when stressed but not when not stressed. This differential effect complicates subjective apprehension of change, but animal and human research clearly document the existence of such forms of learning (e.g., Mazur, 2017; Rachlin et al., 2018). By emphasizing the role of the impulsive system, FA addresses mechanisms that are not easily available for explicit awareness. For example, although the individual may readily be aware of an immediate mood improvement following a specific behavior (e.g., stop reading statistics and instead spend time in the cantina with friends), the effect of repeated episodes with such escape behaviors being reinforced is not easily available to awareness. One reason is that behavior modified by consequences may progress slowly, often over days and weeks, making change difficult to apprehend (e.g., Svartdal, 1991).

A frequent criticism of behavioristic thinking is that it does not recognize cognition and emotions. This criticism is wrong if “behaviorism” is understood as Skinnerian “radical behaviorism” (see Skinner, 1974). FA, being based on the Skinnerian model, regards behavior as a primary datum. But misunderstandings rapidly occur if one does not recognize that “behavior” also includes thinking and feeling. As noted, FA regards feelings and cognition as important in analyzing the control conditions for procrastination. Here, procrastinatory behaviors (i.e., behaviors occurring during planned goal striving that are non-functional in reaching that goal) interact with thinking and feeling in complex ways. However, in FA, these behaviors tend to be dependent variables. Thus, an intention to procrastinate (cognition) is not necessarily the cause of the overt procrastinatory behaviors that follow. We need to understand why the individual decides to procrastinate in the first place. Here, FA provides a well-documented methodological, empirical, and practical approach that explores the complex interplays between antecedents, procrastinatory behaviors, and consequences in establishing and maintaining cognitions and feelings associated with this detrimental habit.

## Conclusion

The present paper is, to the best of our knowledge, the first to systematically explore the utility of functional analysis to the procrastination problem. FA presents a strategy for analyzing controlling conditions for procrastinatory behaviors, and such analyses, in turn, provide powerful information to identify factors that may be changed to prevent and alleviate procrastination. Although FA is compatible with traditional approaches to the procrastination problem, we note that a particular strength of FA is its focus on behavior. As such, FA may add precision to the identification of procrastinatory behaviors and their controlling conditions, providing valuable information for prevention and reduction of procrastination.

## Author contributions

Both authors listed have made a substantial, direct, and intellectual contribution to the work and approved it for publication.

## Funding

Publication charges were covered by the publication fund of UiT The Arctic University of Norway.

## Conflict of interest

The authors declare that the research was conducted in the absence of any commercial or financial relationships that could be construed as a potential conflict of interest.

## Publisher’s note

All claims expressed in this article are solely those of the authors and do not necessarily represent those of their affiliated organizations, or those of the publisher, the editors and the reviewers. Any product that may be evaluated in this article, or claim that may be made by its manufacturer, is not guaranteed or endorsed by the publisher.

## Footnotes

^1^In the present context we focus on the student population. It should be recognized, however, that even though students procrastinate more than people in general population, the principles described here most probably will apply to the understanding of procrastination in general. For example, Steel et al. (2018, p. 12) compared a student sample and a sample from the general population (total *N* = 7,400, of which 2,435 were students) on 10 procrastination-relevant scales (e.g., temptation susceptibility, attentional and emotional distractibility, lack of energy, and planning) and found very similar patterns over the two groups. Similarly, Svartdal et al. (2016) examined two procrastination scales as well as scales measuring temptation susceptibility and psychological wellbeing over six European countries, largely confirming previous findings that students procrastinate more than employees, that men procrastinate somewhat more than women, and that singles procrastinate more than those in a relationship. Again, only minor differences between the student and employee populations appeared. As for procrastination scales, results from multigroup confirmatory factor analyses indicated that cultural differences were small and largely appeared on scale items measuring timeliness.

^2^"Behavior" is given a very broad meaning in FA. The concept includes observable acts (overt behaviors), but any activity that can occur in a living organism would be regarded as behavior.
